# Epitaxial Guidance of Adamantyl-Substituted Polythiophenes by Self-Assembled Monolayers

**DOI:** 10.1021/acsomega.4c04616

**Published:** 2024-09-03

**Authors:** Dominik Farka, Martin Ciganek, Dominik Veselý, Lukáš Kalina, Jozef Krajčovič

**Affiliations:** † Institute of Organic Chemistry and Biochemistry (IOCB), Czech Academy of Sciences, Flemingovo Náměstí 2, Prague 160 00, Czech Republic; ‡ Faculty of Chemistry, Brno University of Technology (BUT), Purkyňova 118, Brno CZ-612 00, Czech Republic

## Abstract

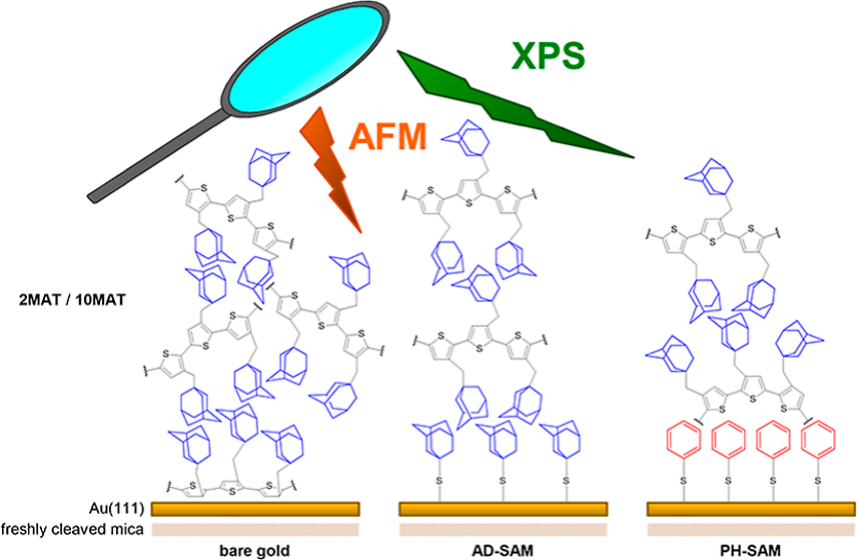

The anisotropic nature of charge transport through organic materials requires high control over the self-assembly of the organic materials. This is particularly so for conductive polymers, where transport occurs mainly along the polymers’ backbone. Herein, we demonstrate the use of self-assembled monolayers (SAMs) to influence the self-assembly of poly(3-adamantylmethylthiophene). We employ two different SAMs, which interact with either the adamantyl- or the thiophene-functionality, respectively, and acquire distinct topologies as compared to the unmodified Au(111) surface. We compare these results with unmodified glass and mica (muscovite) surfaces, which are typically employed in the field of optoelectronics. We prove the usefulness and applicability of epitaxial effects and adamantyl substituents for organic electronics. This presents a viable way toward improved electronic performance for the field as a whole.

## Introduction

Control over the self-assembly of conductive polymers has recently led to outstanding conductivities of 7520 ± 240 S cm^–1^ in poly(3,4-ethylenedioxythiophene) (PEDOT).^1^ This was the culmination of years of diligent research by the Gleason group.^2,3^ Naturally, the question arises whether this success can be repeated in other polythiophenes.

PEDOT possibly presents the best-studied conductive polymer.^4^ Previously, strategies to improve its conductivity involved solution shearing,^5^ acid treatment,^6−9^ strong doping agents,^10^ the introduction of oxidative chemical vapor deposition (oCVD),^2,11,12^ self-doping,^13^ and the creation of nanowires through the use of structures’ surfaces.^14^ Through the latter, the current conductivity record of 8797 S cm^–1^ was achieved. Recently, even approaches without an added oxidant were successfully attempted.^15^ The key to success of PEDOT lies in its proclivity to form intramolecular bonds which stabilize it in a planar conformation,^20^ suppress disorder,^21^ and lead up to mesoscopic 2D transport.^22^ Through efficient disorder suppression, it was possible to achieve temperature-independent transport^12,17,23^ and even magnetic properties in polymers.^12,19,22^

In other polymers, strategies such as molecular orientation through substrate stretching^16^ and functional doping agents such as 2,3,5,6-tetrafluorocyanoquinodimethane (4FTCNQ)^17−19^ or hydrogensulfate^11^ have been applied.

In high-performance organic conductors, a trend became increasingly apparent: a continuous decrease in thickness over the past few years.^1,4,24^ Similarly, the record conductivity was measured in nanowires formed on highly controlled surfaces.^14^ Thus, we arrived at the hypothesis that the epitaxial effects of the substrate play a significant role in material quality.

This hypothesis was confirmed as self-assembled monolayers (SAMs)^25^ were employed to achieve changes in topography and surface potential. PEDOT was grown on different SAMs on gold: one with an adamantyl (AD) headgroup and another one with a thiophene group. Surprisingly, entirely changed topographies and surface potential differences of several hundred mV were observed.

This begs the question, whether this effect can be further enhanced when the substitution of the polymer and the SAM are matched, i.e., can a highly organized adamantane-induced packing be achieved?^26,27^

**Figure 1:**
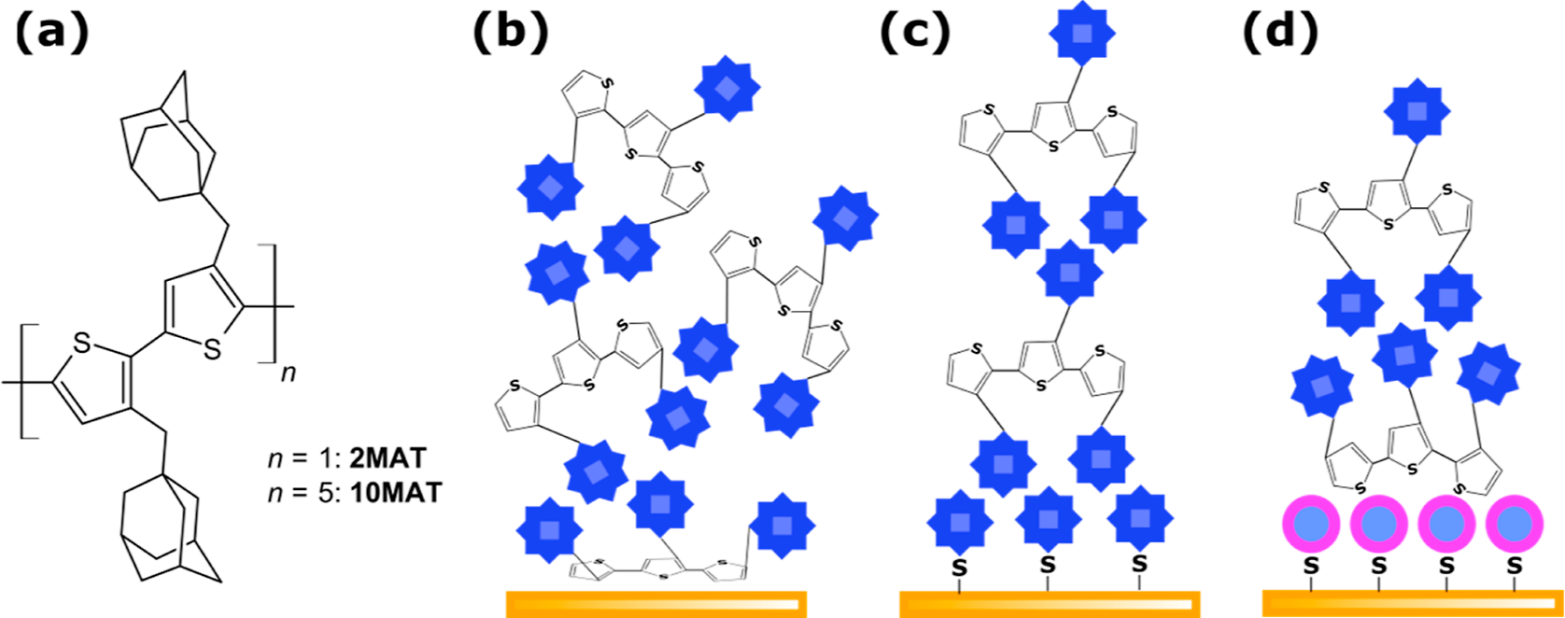
(A) Chemical structure of PMAT and the respective chain lengths for 2MAT and 10MAT. (b–d) Illustration of envisioned epitaxial effects on the self-assembly of PMAT trimers on gold surfaces. Blue structures indicate adamantyl functionality, and pink/blue circles indicate phenyl functionalities. (b) Bare gold, (c) AD-SAM, and (d) PH; a face-on, E-conformer edge-on, and Z-conformer edge-on are expected, respectively.

Therefore, we use the recently published poly(3-adamantylmethylthiophene) (PMAT; Figure 1a) as a model system for our hypothesis.^28^ This material is similar to the conventional poly(3-hexylthiophene) (P3HT) but replaces the hexyl substituent with methyladamantane. These bulky substituents shield the polymer from its surroundings, which becomes apparent when the absorption and emission spectra of the solid and solution are compared. The materials form a granular structure on top of plasma-treated glass and are shown to prefer an edge-on orientation. Through these substituents, the polymers achieve a proclivity to structural order which allows for mobilities in the range of the much smaller P3HT.^28^

In this article, we compare the self-assembly of PMAT (dimers and decamers, 2MAT and 10MAT) on SAMs and observe their self-assembly during spin-coating. We employed flame-annealed gold (111)^29^ surfaces coated with thiols of the following head-groups: adamantyl thiol (AD) and phenylthiol (PH). PMAT was deposited also on bare gold surfaces, mica, and glass to serve as a blank.

Each of these surfaces offers PMAT different modes of interaction and causes alternative self-assemblies. On bare gold, one would expect a face-on configuration, while in the case of AD, an edge-on configuration. In the case of PH, π-stacking is possible; hence, a driving force toward a fully Z-conformation of the polymer backbone may form (Figure 1).

We observe changes based on polymer length and spin-coating conditions. The decamers appear to have limited solubility, and large clusters are immobilized on the AD surfaces. This led us to believe that the adamantyl functionalities cover the surfaces of these structures. We conclude that different modes of interaction exist and propose a way forward for this new polymer.

## Methods

### Material Synthesis and Characterization

PMAT-based oligomers were synthesized as reported elsewhere.^28^ All solvents and reagents were obtained commercially and used as received, unless stated otherwise. All moisture-sensitive reactions were performed in a dry glass apparatus under a positive pressure of argon. Solvents used for the purpose of purification were obtained from Penta Chemicals (Czech Republic) in p.a. grade. A total of two major fractions were obtained: a dimer (2MAT) as a yellow-brown solid material, yield 37 mg/12.4% and a decamer (10MAT) as a red solid material, yield 54 mg/18.2%, mp >330 °C. ^1^H NMR (CDCl_3_, TMS): δ 6.92 (br s, 1H), 2.65 (br s, 2H), 2.38 (br s, 1H), 1.88 (s, 4H), 1.58–1.11 (m, 19H), see Figure S1.

^1^H NMR spectra were recorded on a Bruker AVANCE III 500 MHz FT-NMR spectrometer in CDCl_3_. Chemical shifts (δ) are given in parts per million (ppm) relative to TMS as an internal reference. FTIR spectra were recorded on a Bruker ALPHA II compact FT-IR spectrometer using the ATR-FTIR method with a diamond crystal. The melting point was determined on a Kofler apparatus, and the temperature was not calibrated.

The measured polymer parameters were as follows: number-average molecular weight (*M*_n_/g mol^–1^), weight-average molecular weight (*M*_w_/g mol^–1^), and dispersity. All the polymer properties were measured via a GPC system (Agilent 1100, Santa Clara, CA, USA) in chloroform (CHCl_3_), and the analysis parameters were as follows: mobile phase flow, 1 mL min^–1^; column temperature, 23 °C; used column, PLgel 5 μm MIXED-C (300 × 7.5 mm).

### Thin Layer Preparation

Gold films were evaporated in UHV via physical vapor deposition (PVD) on freshly cleaved mica and flame-annealed to form atomically flat Au(111). To form SAMs, ethanoic solutions of the relevant thiols were prepared according to established procedures.^25^

PMAT solutions of 1 mg mL^–1^ in chloroform were prepared and used as in the case of 2MAT. For 10MAT, they were filtered (0.45 μm PVDF filter) prior to use to remove conglomerated particles. Films of PMAT derivatives were formed via spin-coating 30 μL of oligomer solution using “slow” (6 s at 500 rpm; 30 s at 1250 rpm) and “fast” (6 s at 500 rpm; 30 s at 1500 rpm) recipes. The preparation of drop-cast films was abandoned, as the epitaxial effects were masked by large quantities of the deposited material.

### AFM Measurements

All measurements were done on a Bruker Multimode AFM/STM system with SNL-10 AFM tips (cantilever D) from the same company. All measurements presented were performed in the “ScanAssist HR in Air” mode. Each type of material was measured with a fresh tip, and contamination of 2MAT samples with 10MAT can be ruled out, and vice versa. All mechanical measurements were performed in order to gain a differential image of individual features; hence, all mechanical values are in arbitrary units.

All measurements presented were performed in the “Peakforce HR in Air” mode. All thickness measurements were performed by “nanoshaving” (contact mode with the maximum setpoint applied to scratch off any material above the gold layer) an area clear of any material and subsequent topography assessment as described above.

### Angle-Resolved XRD

Angle-resolved X-ray diffraction (XRD) measurements were performed in an Empyrean (PANalytical) diffractometer according to the literature.^25^ Due to the strong signal of the substrate, any signal emerging from the substrate was masked. A detailed explanation is given in the Supporting Information.

### XPS Measurements

X-ray photoelectron spectroscopy (XPS) analyses were carried out with an Axis Ultra DLD spectrometer using a monochromatic Al Kα (*h*ν = 1486.7 eV) X-ray source operating at 75 W (5 mA, 15 kV). The spectra were obtained using an analysis area of ∼300 × 700 μm. High-resolution analyses were measured with the step size of 0.1 and pass energy of 20 eV. The Kratos charge neutralizer system was used for all analyses. The instrument base pressure during the measurements was consistently at 2·× 10^–8^ Pa. From the point of view of SAM’s characterization, angle-resolved XPS (ARXPS) was chosen as a nondestructive method to determine the physicochemical properties. The take-off angle, the angle of the sample with respect to the direction probed by the detector, was set to 45°. The method for measuring the thickness and surface coverage of SAMs from ARXPS data was adopted based on Cumpson’s^30^ thickogram design. The spectra were analyzed using CasaXPS software (version 2.3.17) and have been charge-corrected to the main line of the carbon C 1s spectral component (C, C, H) set to 285.0 eV.

## Results and Discussion

On the three types of surfaces on gold (AD, PH, and bare Au 111), the different proclivities for self-assembly unfold in PMAT (Figure 1b–d). On bare gold (Figure 1b), π-electrons may interact with the metallic surface; consequently, the polymer is expected to chemisorb in the face-on orientation, which may lead to an increase in disorder. In the case of AD-SAM (Figure 1c), an epitaxial effect is expected to favor self-assembly in the Z-conformation. At last, the deposition on PH-SAM (Figure 1d) offers the polymer the possibility to π-stack directly with the surface; an E-conformation may be therefore adapted as opposed to AD, where the Z-conformer will form, exclusively. We do not exclude the possibility of nonaromatic interactions in the case of PH-SAM.

Further, since previous studies on elongated chains of PMAT showed crystalline organization, the SAM may modulate this in 10MAT. Further, the longer chain length will impose a directionality of growth on the contrary to 2MAT, which will behave as a small molecule. A slower angular velocity in spin-coating is expected to give the molecules more time to adhere efficiently to the surface and be closer to their thermodynamic minimum. However, the poorly dissolved material will have a higher chance to be immobilized, where a strong interaction with the surface is possible.

### X-ray Photoelectron Spectroscopy

**Figure 2:**
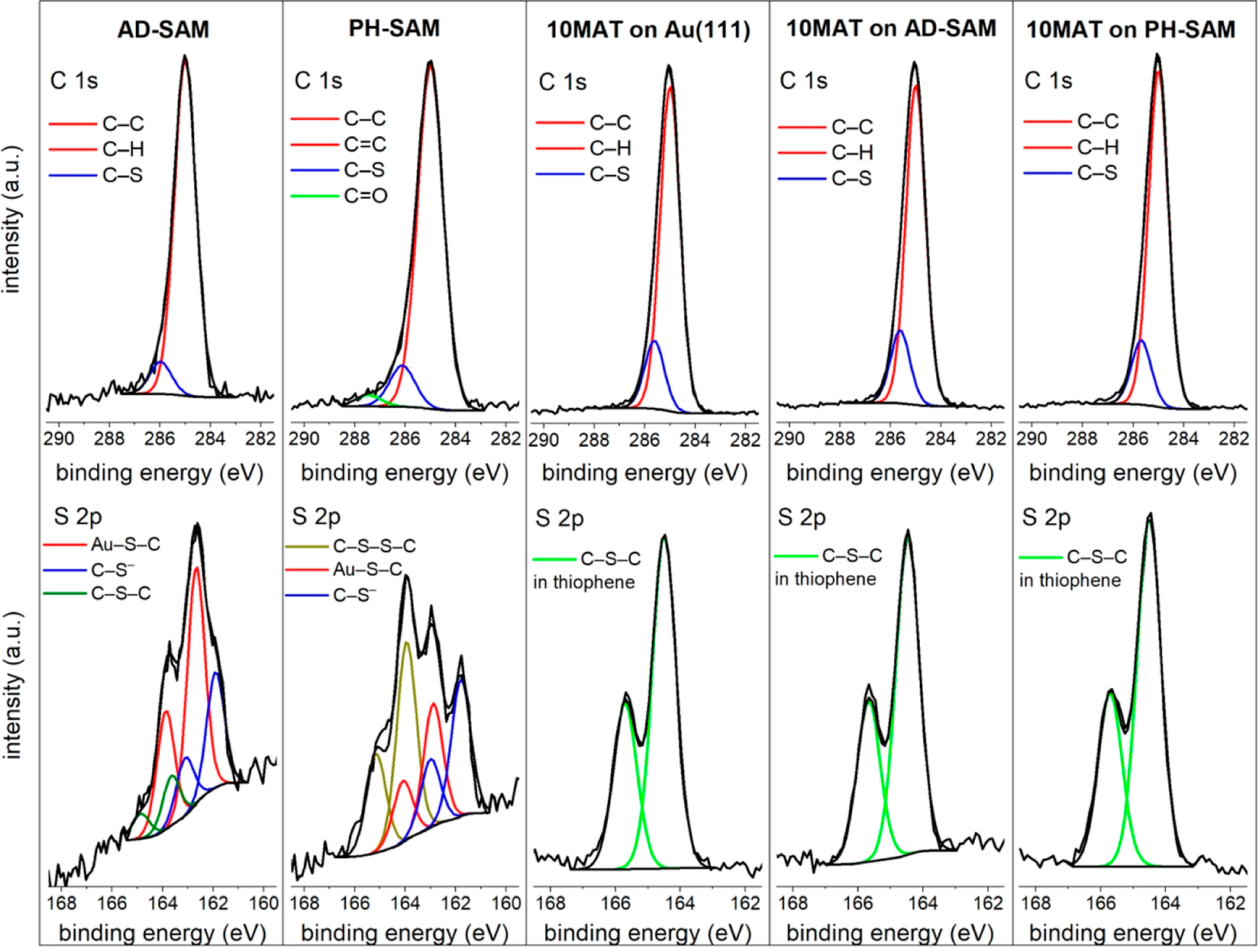
XPS measurements of AD- and PH-SAM and 10MAT on three different surfaces which give identical signals. Therefore, additional characterization of oligomer-covered surfaces was abandoned.

The SAMs were analyzed by XPS, especially in terms of S 2p and C 1s high-resolution spectra (Figure 2). The S 2p spectra acquired for SAMs have a typical doublet structure due to the spin–orbit splitting characterized by the S 2p_3/2_ and S 2p_1/2_ peaks. All S 2p spectra were fit using the 2:1 peak area ratio and 1.2 eV splitting. In the case of AD-SAM, the S 2p spectrum was deconvoluted to three components. The binding energy for S 2p_3/2_ at 162.7 eV corresponds to the presence of sulfur from the adamantyl thiol group directly bonded to the gold (111) substrate.^31^ According to the semiquantitative analyses, the proportion of sulfur in the Au–S–C bond within AD-SAM is approximately 60 at. %. The second most abundant chemical state of sulfur shows the S 2p_3/2_ component at 161.9 eV. In agreement with Laibinis et al.,^32^ this corresponds to the monolayers of thiolates adsorbed to the gold surface. In our measurements, this chemical state constitutes 30 at. % of the monolayer. The remaining 10 at. % of the S 2p_3/2_ peak at 163.7 eV can be explained either as C–S–C bonds^33^ or as disulfides (Δ*E* = 0.3 eV).^34^ Given the chemical environment and the chemical history of the samples, we lean toward the presence of physisorbed disulfide species. The C 1s high-resolution spectrum shows only two peaks associated with the C–C; C–H bonds (285.0 eV) and C–S bonds (286.0 eV).

For PH-SAM, a similar situation was found as that for AD-SAM. A direct bond to gold (S 2p_3/2_ component at 162.9 eV) and a thiolate species (S 2p_3/2_ at 161.8 eV) were both detected and made up 27 and 30 at. %, respectively. The largest portion of the sulfur species (43 at. %) was made up of the S 2p_3/2_ signal at 164.0 eV. This corresponds to physisorbed disulfide molecules.^34^

The deconvolution of the C 1s spectrum of PH-SAM gave three peaks. The main peak at 285.0 eV corresponds to the C–C; C–H groups and also contains a contribution of the sp^2^-hybridized carbon in aromatic rings. The second peak at 286.1 eV points to the C–S bond. The third peak at 287.5 eV corresponds to C=O groups (surface contamination). The subsequent application of angle-resolved XPS on both SAMs reveals their thickness (*d*) and surface coverage (Γ).^35,36^ The results are shown in Table 1.

**Table 1:** Properties of PMAT-Based Oligomers Measured via GPC

MAT fractions obtained	*M*_n_/g mol^–1^	*M*_w_/g mol^–1^	dispersity [-]	approx. number of units [-]
2MAT	400	581	1.45	2
10MAT	552	2613	4.73	10

The thickness of AD-SAM was about 1 nm, which corresponds to the upright-standing material chemisorbed to gold (Table 1).^37^ The situation in PH-SAM (thickness = 2.6 nm; Table 1) was, again, more complex. The measured values are significantly thicker than what is expected of pristine thiols or thiolate films or even physisorbed disulfide species coordinated to gold through single sulfur. The best explanation therefore is the presence of a bilayer which consists of a chemisorbed monolayer of thiols and thiolates, which allows for the physisorption of disulfide species as a second monolayer as well as oxidized phenylthiol or similar impurities. Given the calculated surface coverage of both surfaces, the ratio of 1:4 in favor of PH would be reduced to 1:2 and is in good agreement with the coverage ratio expected due to the geometric differences between both molecules (Table 1).

XPS analysis was also applied to thin-film layers of PMAT (10MAT) deposited on the SAM surface. Regardless of the substrate used, the chemical composition was the same in all cases. The S 2p high-resolution spectra show the bond between carbon and sulfur in the thiophene ring (S 2p_3/2_ at 164.5 eV),^38^ and the C 1s spectra refer to the presence of C–C; C–H (285.0 eV) and C–S bonds (286.0 eV) in the polymer chain.

### Atomic Force Microscopy

**Figure 3:**
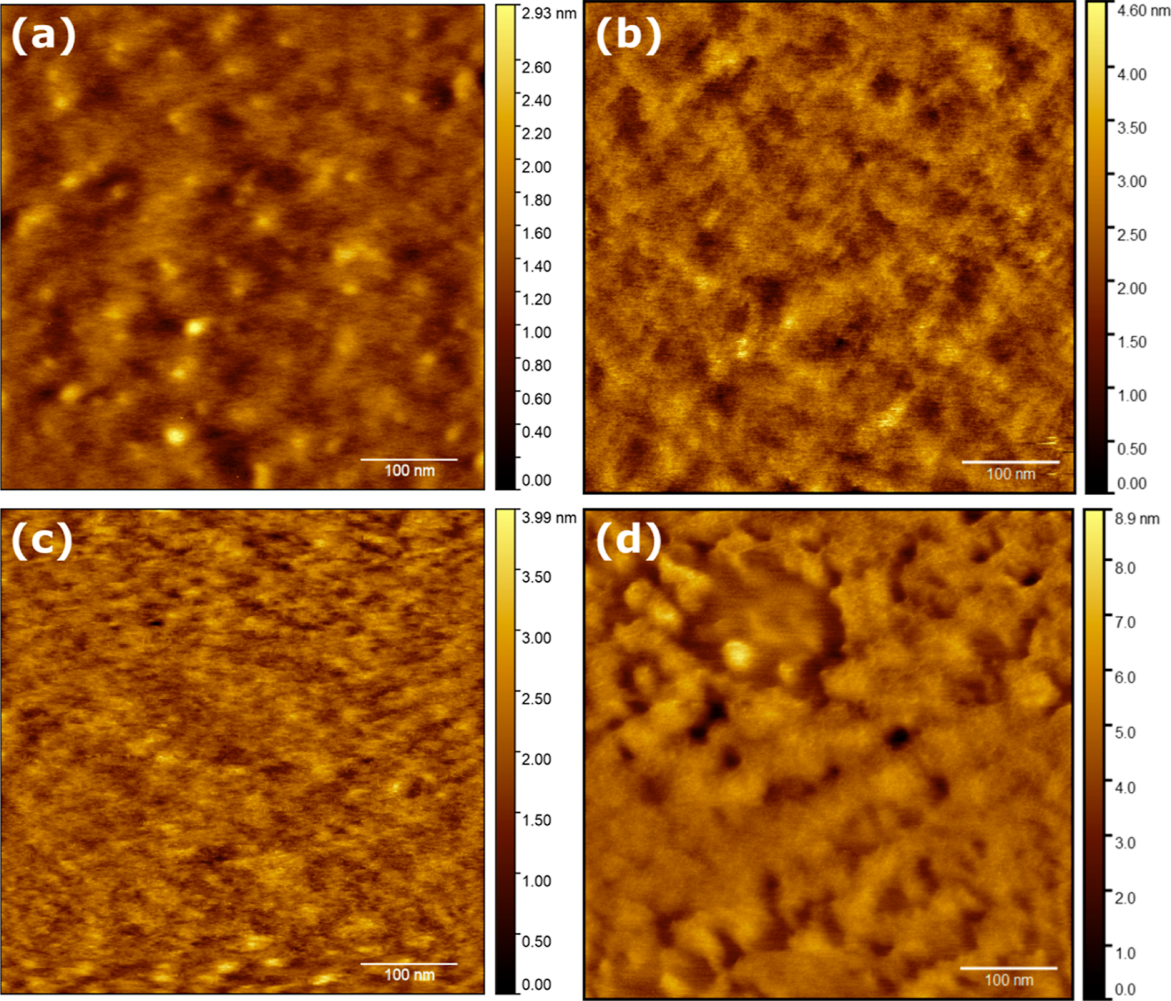
Bare Au. 2MAT deposited via (a) slow and (b) fast recipes. 10MAT deposited at slow (c) and fast (d) recipes.

On bare gold (Figure 3), smooth films of fibrous or finely granular material were achieved with respect to thickness variation and root-mean-square (rms) roughness. For 2MAT (Figure 3a,b), the films showed rms roughness values of 0.269 and 0.501 nm for the slow and fast procedures, respectively, which are in good agreement with the average rms (Table 2). Tens of nanometers large flat isolated areas formed separated by rough elevated structures, some of which formed peaks or spires. These towered over the surrounding area at a relative height of 2 nm and above. The respective thicknesses were determined by atomic force microscopy (AFM) measurements as 6.5 and 2 nm, respectively, For 10MAT on bare gold (Figure 3c,d), a directionality in growth was observed with the rms roughness values of 0.374 and 0.888 nm for slow and fast rotation speeds, respectively. The average rms roughness showed a significant variation (Table 2). Also here, the thicknesses decreased with the spinning speed from 24 to 16 nm, respectively. For slow speeds (Figure 3c), a wave-like pattern was observed. Within a few hundred nanometers, the organization of the material changes, indicating no long-range propagation of assembly. In the top part, a pattern of deep and high areas similar to the one for 2MAT is observed, while in the central part, the depressions get fewer, though larger and shallower. On the lower edge of the image, the highest peaks are concentrated. This indicates a different, faster mode of self-assembly and crystallization being present in that area. At faster spinning speeds (Figure 3d), a pattern of assembly similar to that in Figure 3b was observed, yet less expressed. Further, a tip change occurred after the encounter with the elevated position at about one-third of the image (scanning direction, top-to-bottom). As resolution was lost, we interpret this as the picking up of material from the surface, which indicates a weakened intramolecular attraction between individual chains.

**Table 2:** Thickness and Surface Coverage of SAMs Calculated from XPS Data

	*d* (nm)	Γ (10^14^ mol cm^–2^)
AD-SAM	1.0	3.7
PH-SAM	2.6	15.4

**Figure 4:**
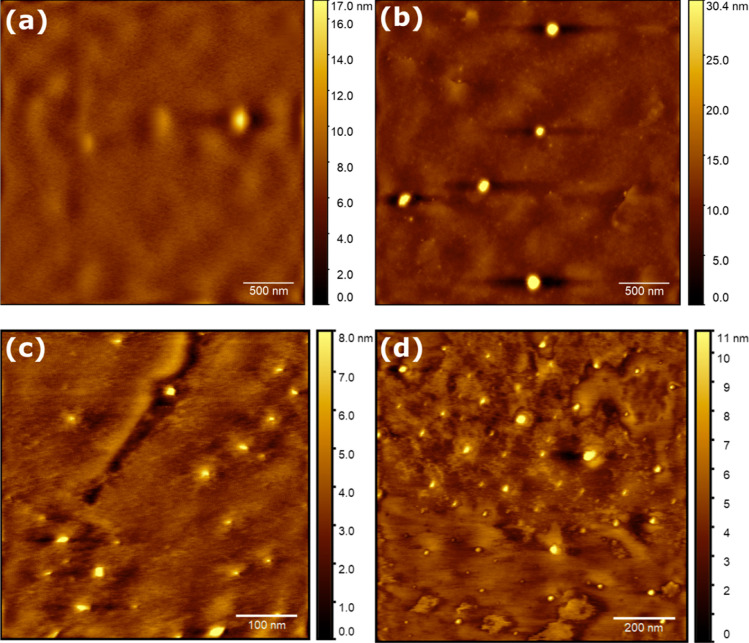
AD-SAM. 2MAT deposited via (a) slow and (b) fast recipes. 10MAT deposited at slow (c) and fast (d) recipes.

The samples deposited on AD-SAM (Figure 4) were significantly more varied in roughness (Table 2). This is a consequence of large particle-like features found on all surfaces. These tower tens of nanometers above the prevalent material and were initially interpreted as poorly dissolved materials, which remained despite filtration. This was confirmed through the presence of the Tyndall effect (Figure S3). This remained weakly expressed even after the filtration of cooled material solutions (Figure S4).

The thickness of samples on the other hand was significantly more narrowly distributed as compared to that of bare gold (Table 3), which indicates facilitated assembly on the surfaces through the adamantyl functionality.

**Table 3:** rms Roughness Averages and Standard Deviations Thereof

	rms roughness average ± standard deviation			
	2MAT		10MAT	
	slow	fast	slow	fast
bare gold	0.41 ± 0.20	0.58 ± 0.09	0.50 ± 0.17	1.29 ± 0.47
AD	1.09 ± 0.26	1.25 ± 0.9	1.17 ± 0.27	1.28 ± 0.15
PH	5.21 ± 0.33	5.31 ± 0.33	3.22 ± 1.91	2.28 ± 2.00
mica	4.27 ± 3.19	1.37 ± 0.17	4.02 ± 2.09	1.55 ± 0.41

Given the presence of these particles on AD surfaces only, we can deduct the presence of the very same adamantyl functionalities on their surfaces. Likewise, we can rule out the presence of functional groups with strong interactions with gold or PH (i.e., thiophene functionalities).

For 2MAT on AD-SAM (Figure 4a,b), rms roughnesses of 0.954 and 2.537 nm for the slow and fast recipes were observed, respectively. The rms roughness among samples varies strongly due to the particles present (Table 2). However, when the immobilized particles were excluded from the roughness calculation (Figure 4b), the rms roughness decreased to 0.953 nm in the latter material. Consequently, we conclude that the employed SAM governs the self-assembly, while the spin-coating recipe plays a minor role and is reflected in the observed thicknesses (Table 3), albeit the final pattern observed may vary in different parts of the sample (Figure S5).

For 10MAT (Figure 3c,d), rms roughness values of 1.042 and 1.279 nm were observed for the slow and fast recipes, respectively, which are in good agreement with the average values (Table 2). Just as before, despite the filtration of the mother solutions, colloidal materials were immobilized on the surfaces during deposition. Since the overall heights were lower than that in the case of unfiltered 2MAT, it appears that the filtering procedure removed larger particles; smaller particles passed through. Also, in this case, similar thicknesses were observed for the two recipes with thinner films for faster spinning speeds (Table 3).

**Figure 5:**
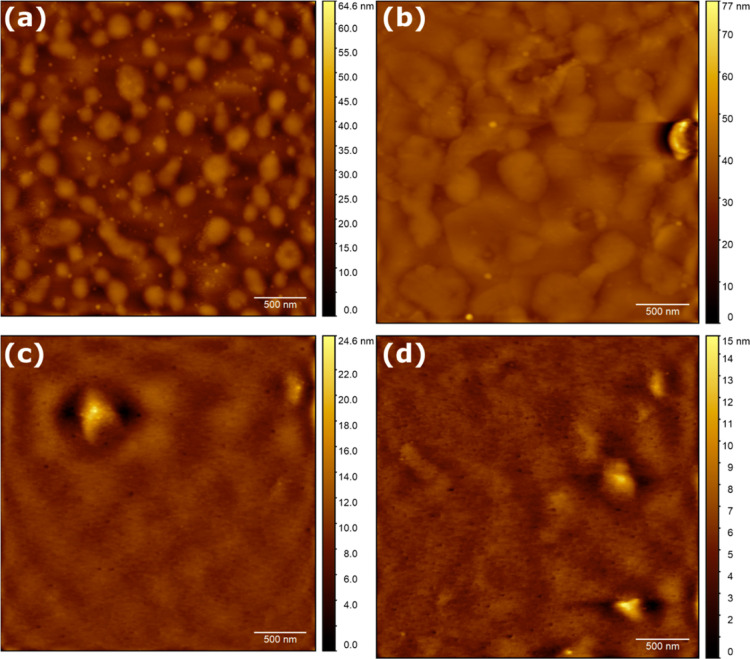
PH-SAM. 2MAT deposited via (a) slow and (b) fast recipes. For both samples, the absolute majority of structures had a height distribution of 30 and 25 nm, respectively. 10MAT deposited at slow (c) and fast (d) recipes.

For PH-SAM (Figure 5), we expected an initial layer of Z-conformed PMAT to form, covered in a more concisely arranged polymer material. On the contrary, significantly rougher films were observed, in particular for 2MAT.

In the 2MAT films (Figure 5a,b), the rms roughness values of 4.793 and 5.546 nm were observed for the slow and fast recipes, respectively. Despite the higher roughness values as compared to AD-SAM and bare gold, both 2MAT recipes gave narrowly distributed rms roughness values (Table 2). On the other hand, faster-spun samples resulted in thicker films (Table 3). This trend reversal indicates a difference in the growth mechanism for particular areas. Once a particular (crystal) growth mode is achieved, it starves its surroundings of novel material. Further, two types of elevated features can be found: large, hundreds of nanometers in area plateaus and tens of nanometers high, needle-like spires towering above the bulkier structures. The differences between both the structures will be discussed later in this paper. Similar structures, albeit less frequent, were found when the samples were spun at faster speeds with 2MAT.

For 10MAT (Figure 5c,d), a similar reversal in trend was observed with rms roughness values of 1.238 and 0.861 nm for slow and fast spinning speeds, respectively. In contrast to that of 2MAT, these values varied largely between measurements (Table 2). Also, none of the spire-like structures were observed; hence, this type of growth is linked to a lower molecular weight and may require the flexibility and modularity of a smaller starting material such as the quick adaptation of Z-conformation as a single monomer–monomer bond can be rotated much more efficiently at a lower energetic cost rather than a fivefold larger structure. Both varieties of deposited 10MAT included a few elevations significantly higher than those of their surroundings. These were larger than the structures found for 2MAT and also less concisely defined and, hence, appeared more blurred. With regards to film thickness, we observe a return to an inversely proportional relationship between the spinning speed and film thickness (Table 3). In the light of XPS measurements, this indicates the necessity of higher shear forces for the removal of physisorbed disulfides, which are replaced by the Z-conformer of 2MAT, lest serendipitous effects were involved.

## Discussion

From the measured AFM images obtained, it becomes apparent that interactions via largely available moieties, such as through the polymers’ π-electrons with gold (Figure 3), or unspecific interactions on amorphous glass (Figure S8) result in small rms roughness values but also poor intermolecular attractions which manifest in tip changes (Figures 3d and S6b, both in the lower part).

The engineered adamantyl–adamantyl interaction (i.e., adamantyl-induced packing) present on AD-SAM is possibly the hardest to interpret. Typically, an inverse relationship between the spinning speed and film thickness is expected, while very similar values were observed for this material. Additionally, the presence of large conglomerates which were proven to be colloids of poorly dissolved polymers in the mother solutions further shrouds the image. Given the known tendency of PMAT to form the crystalline features of polymers in the E-conformation, such conglomerates are covered in the necessary adamantyl functional groups. In general, however, the material formed neat, smooth films, and these larger features are excluded. This gives hope that AD-SAM may be used in application once conglomeration can be controlled. Two possible approaches consist of the reduction of concentration or the additive of anticoagulants (or the change of solvent) in the solutions. An entirely different approach to achieve this may lie in the avoidance of the solution phase entirely. An elegant bypass might lie in the application of oCVD.^11,25^

However, in the parts where fewer, or none, of the conglomerates were immobilized, a netlike structure became apparent (Figure S5). These were only about 3.5 nm in height, which corresponds to the vertical height of the polymer, as expected from the literature (3.5–3.7 nm).^28^ Consequently, these are elevations caused by the polymers in the E-edge-on^1^ conformation relative to the substrate. A similar structure was found in the samples deposited on mica (Figure S6). Here, multistep deposits were found, which led to apparent pockmarks on the substrate. In 2MAT, we were able to observe four layers (Figure S6a) and three distinct layers (Figure S6b), for slow- and fast-spun films, respectively. For 10MAT, the achieved topologies show more steps as more elevated structures attract more of the dissolved material.

Possibly, the most complex and most surprising behavior was found in layers deposited onto PH-SAM. In contrast to AD-SAM, an edge-on conformation of the initial layer is only possible where the first PMAT layer is in Z-conformation. A chain growth perpendicular to the surface, such as that reported previously,^25^ is hypothetically possible and would result in a topographically isolated, spire-like growth away from the surface. These two types of growth might differ in electronic and mechanical behaviors alike.

The first difference with that of the other gold surfaces was that slowly spun samples were rougher and also thinner. This indicates a preferred deposition in some areas over others, i.e., kinetically controlled growth. This may also be related to the necessity to remove physisorbed SAM constituents prior to deposition. In light of the above statements on the formation of an initial layer, this is of little surprise. However, this is only found for 2MAT, which is more flexible, and only a single monomer–monomer bond has to be rotated to form the initial layer. In 10MAT, the formed structures are larger, flat, and rather uniform in height, safe for a few exceptions. These few exceptions are possibly the results of the adaptation of a similar first layer to that in the broader structure found in 2MAT deposited on PH-SAM.

The said combination sparked particular interest in us as it showed two types of features: broad elevated plateaus and concise spires that tower over the surface topography. We therefore measured the mechanical properties by AFM. In this way, we were able to gain more information from individual features, as shown for 2MAT deposited on glass (Figure S7).

**Figure 6:**
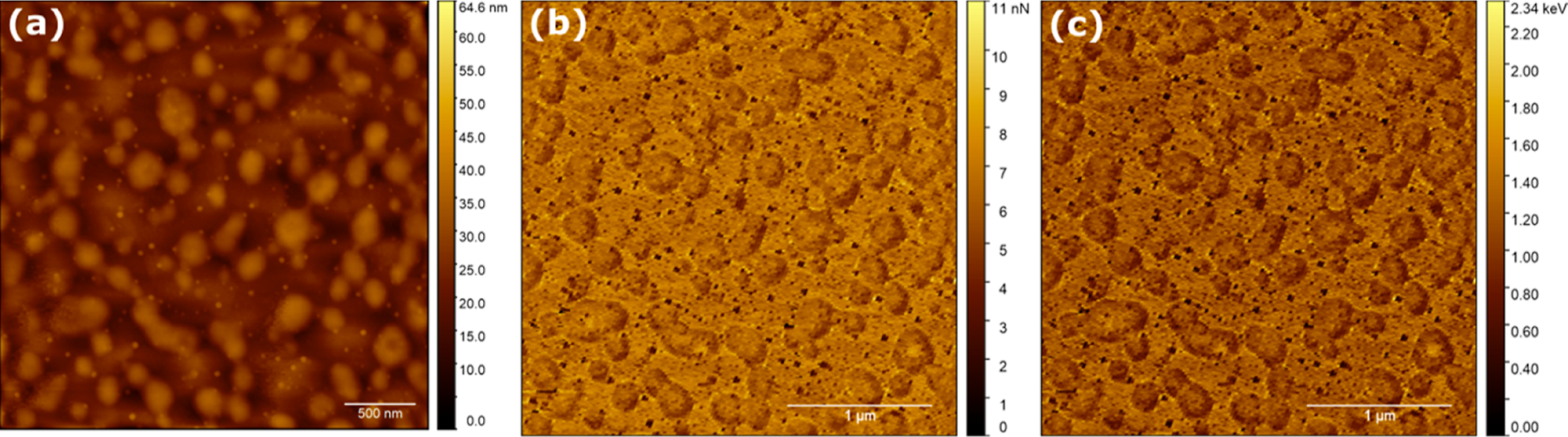
Imaging of mechanical properties of PH-SAM. (a) Topography, (b) adhesion, and (c) dissipation image of the same area. Color scale of mechanical experiments in arbitrary units.

The topology image (Figure 6a) shows broad circular or elliptical features distributed on a plane. In between these features, small, spire-like protrusions are found that appear different. In the adhesion image (Figure 6b), the said spires show no adhesion toward the AFM tip, while the plain they grow from shows a strong adhesion, similar to the large plateaus. This was confirmed in the dissipation image (Figure 6c), where essentially all of the mechanical energy of the AFM tip impacted the spires in an elastic manner. On the contrary, the peaks of larger structures and the lower lying features of the plain showed a significant dissipation of mechanical energy as both features underwent plastic (permanent) deformation.

## Conclusions

We compared the deposition of PMAT at two distinct chain lengths on the selection of conventional and SAM-covered surfaces with distinct properties. We demonstrate the strong epitaxial impact of some of the SAMs and confirm a layer-by-layer deposition for AD-SAM induced by the adamantyl functionality. At the same time, we observe the physical limitations of the current experimental techniques, which became apparent when angle-resolved XRD was attempted.

We investigated the SAM properties via XPS and observed a mix of both chemi- and physisorbed materials, which resulted in a bilayer for PH-SAM. This further highlights the necessity for a deeper understanding of SAMs to improve the state of the art in organic electronics.

The AD-SAM shows a particularly strict proclivity toward edge-on deposition of the E-conformer which is neatly visible in the formation of step-like levels of 3.5 Å, the polymer height. Once the challenge of conglomeration of material in solution is overcome, this can become an invaluable asset for organic electronics, as a pure edge-on configuration is preferred for charge transport over a mix of configurations.^1^

The most interesting is the kinetically driven film growth on PH-SAM. Its aromatic nature has proven to prevent or limit deposition at initial concentrations. Consequently, the rate-determining step of film growth is the formation of the initial layer. For 2MAT, it was further possible to identify two different structures, broad plateaus and needle-like spires, whose mechanical properties are in stark contrast. The spires show very little adhesion to the AFM tips and are mechanically highly robust. Due to the limited prevalence in 2MAT and its entire absence in 10MAT, we consider that in these, the initial layer consists of a PMAT dimer in Z-conformation.

This work demonstrates the importance of SAMs beyond simple adhesion layers and opens the mind for future adaptations and pathways of investigation for polythiophenes and organic electronics in a broader sense (Table 4).

**Table 4:** Thicknesses of the Investigated Surfaces Measured on Individual Samples via Nanoshaving

	thickness/nm			
	2MAT		10MAT	
	slow	fast	slow	fast
bare gold	6.5	2	24	16
AD	11	12	22	18
PH	2	17	12	7
mica	5	14	20	30

## Supporting Information Available

^1^H NMR; FTIR PMAT (2MAT and 10MAT); Tyndall effect (hot-filtered); Tyndall effect (additionally cold-filtered); AFM images of AD-SAM + 2MAT alternative surface area; AFM images of bare mica; and AFM images of 2MAT on bare glass (topography and mechanical properties) (PDF)
