# Supplementary material for: Epitaxial Guidance of Adamantyl-Substituted Polythiophenes by Self-Assembled Monolayers
Source: ACS Omega. 2024 Sep 3;9(37):38733–42. doi: 10.1021/acsomega.4c04616 (PMC11411674; doi:10.1021/acsomega.4c04616)
Supplement: Supplementary file 1 — ao4c04616_si_001.pdf [file ao4c04616_si_001.pdf]

Supporting Information to:

Epitaxial guidance of Adamantyl-Substituted Polythiophenes by Self-Assembled Monolayers

Dominik Farka* [a], Martin Ciganek [b], Dominik Veselý [b], Lukáš Kalina [b], Jozef Krajčovič [b]

[a] Institute of Organic Chemistry and Biochemistry (IOCB), Czech Academy of Sciences; Flemingovo Náměstí 2, 160 00 Prague, Czech Republic.
[b] Faculty of Chemistry, Brno University of Technology (BUT), Purkyňova 118, CZ-612 00 Brno, Czech Republic
* corresponding author (Dominik.farka@uochb.cas.cz)

Characterization of Oligomers 2MAT and 10MAT

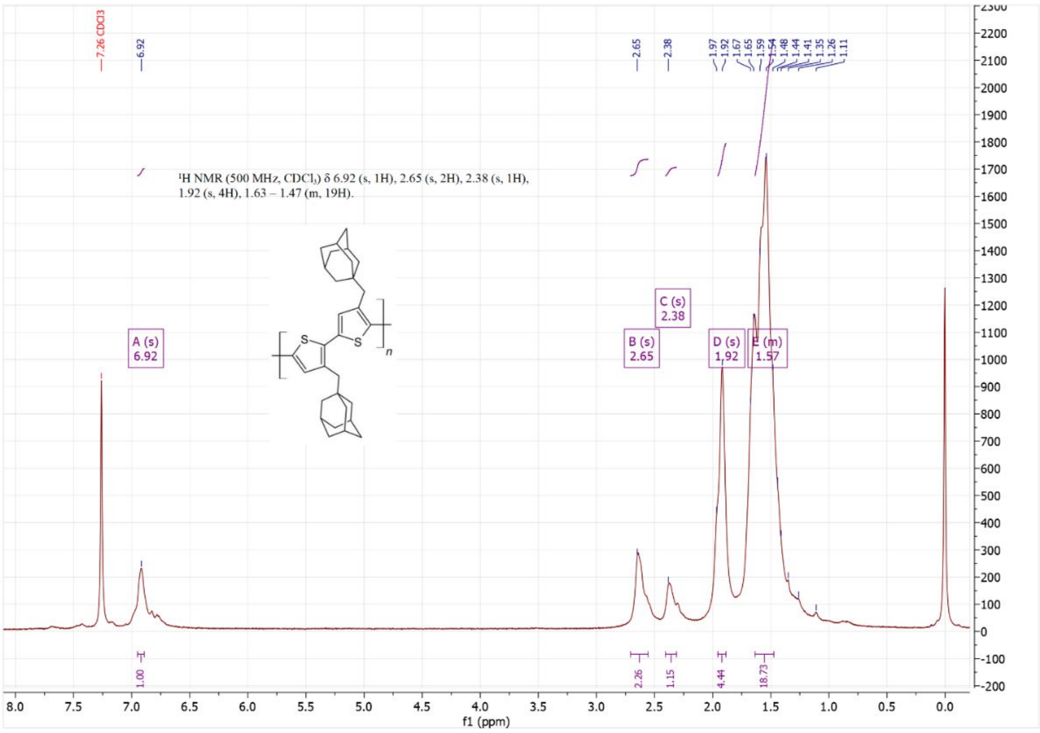

Figure S1: ^{1}H NMR spectrum of PMAT derivative in $CDCl_3$ as solvent.

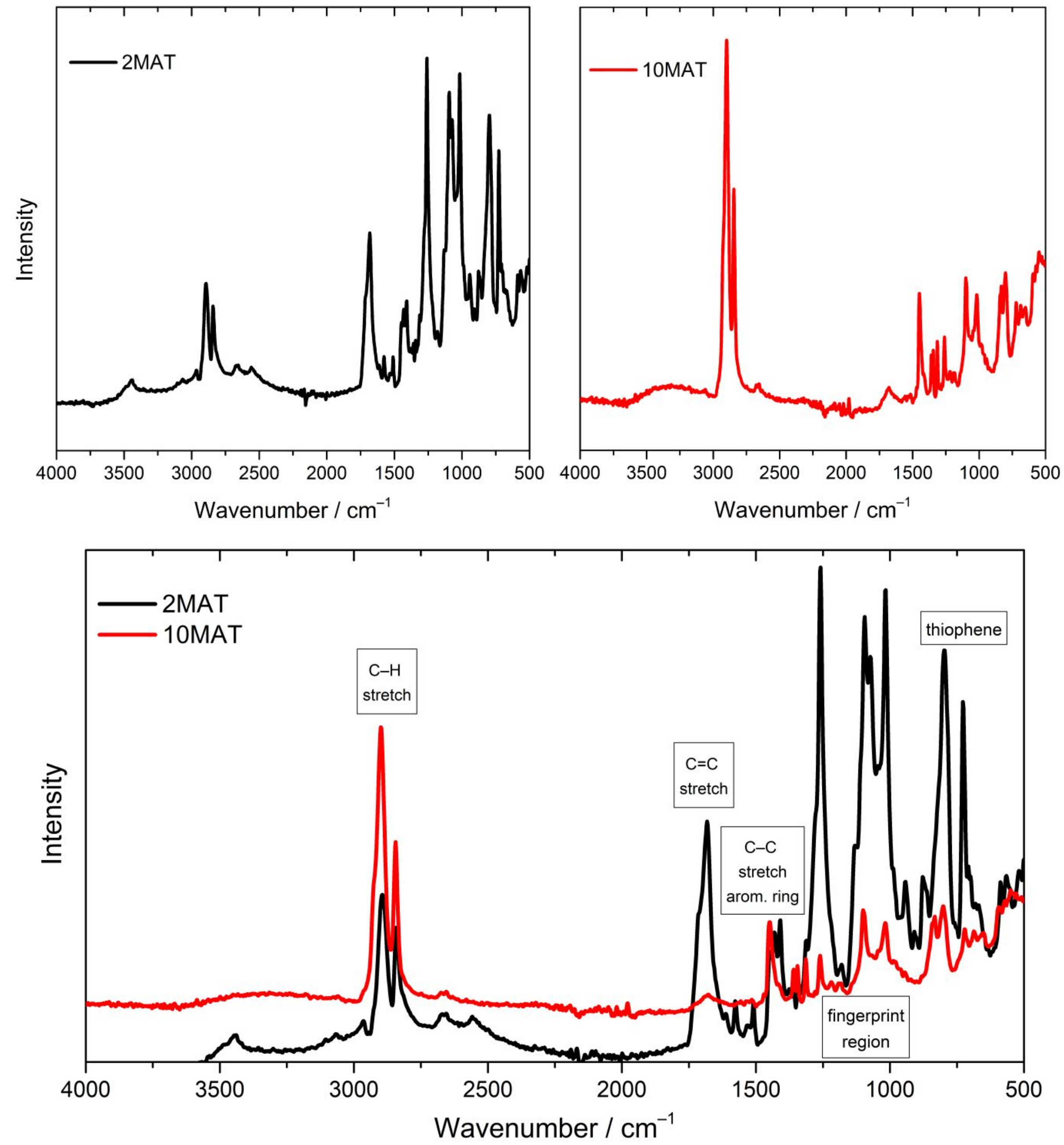

Figure S2: FTIR spectra of 2MAT and 10MAT derivatives. The merged spectrum (in the bottom) clearly shows a higher signal intensity for the 10MAT material compared to the 2MAT in the region of 3000-2850 cm^{-1} indicating C–H stretching in the alkane. This proves the higher content of C–H bonds of the aliphatic substituent (adamantylmethyl group in *β*-position of thiophene) in the decamer (10MAT) compared to the dimer (2MAT).

Optical Measurements to Determine the Tyndall Effect

Large agglomerates were observed in some of the AFM measurements. To determine their origin as stemming from colloidal agglomerates in the solution, we set out to determine this via the Tyndall effect.

We heated the chloroform-solutions/-colloids to 40-45°C and filtered these while hot (0.45 µm PVDF filter). We cooled these solutions to room temperature (22°C) and observed a very distinct Tyndall effect in this case (Fig. S3). Hence, hot-filtered solutions resulted in the presence of colloids.

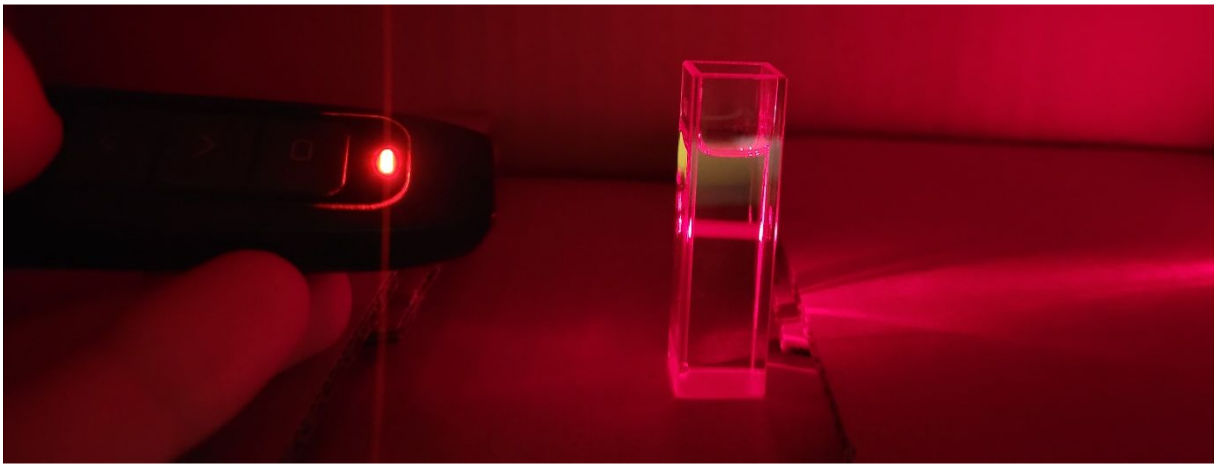

Figure S3: Hot-filtered colloid of shows distinct Tyndall effect.

Afterwards, the same solution was cooled to 10-15°C and filtered again (0.45 µm PVDF filter). After equilibration to room temperature, the Tyndall effect was still observed, yet strongly reduced in intensity (Fig.S4).

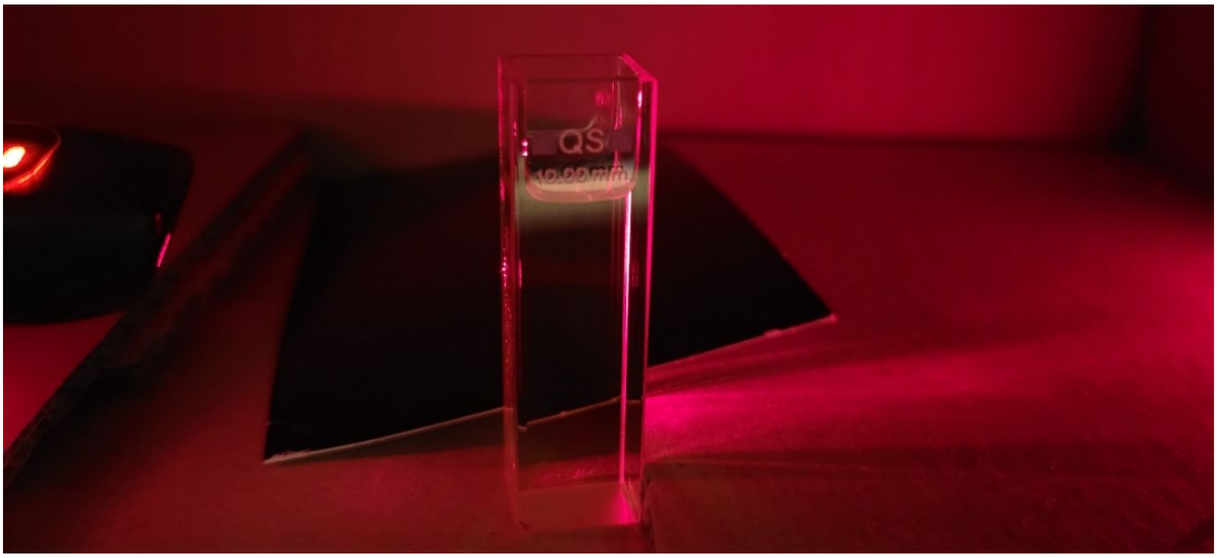

Figure S4: Colloidal dispersion shown in Fig.S3 after cold-filtration. The Tyndall effect is highly reduced, yet remains.

XRD measurements of samples

The X-ray diffraction (XRD) method is an effective tool for determining the structure of a thin polymer film. Unfortunately, during the measurement of our samples, a significant preference orientation of both the substrate and the gold layer was revealed. This was manifested in a very high intensity of (002) planes in the case of mica and (111) planes in the case of gold. Consequently, the determination of crystalline properties including the size of features by means of the Scherrer formula was beyond the analytical strength of this technique.

2MAT on AD-SAM

An alternative surface assembly was observed in the case of AD-SAM (Fig.S1). A boundary between the surface assembly presented in the main paper (Fig.3b) was found. Since this effect is found on multiple heights, we rule out an effect caused by an etched gold substrate. A similar assembly was

found on bare mica (muscovite), albeit the holes formed on AD-SAM are at least double in size and up to quadruple. Consequently, we interpret this as a disordered phase of PMAT self-assembly.

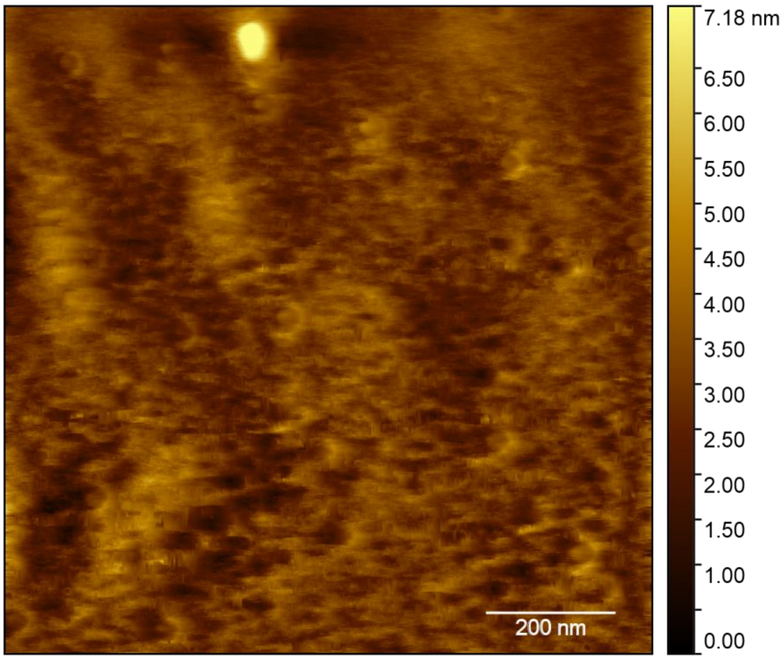

Figure S5: AD-SAM + 2MAT (fast) alternative pattern. Side by side of the archipelago-like structure a "coffee-stain"-like structure emerges. Each pattern was observed to continue in the respective direction for several micrometers, hence we interpret this as a surface initially covered in undissolved material. As the spinning speed increased, the particles were swiped off the surface.

Bare mica

All structures on mica showed a similar pock-mark topography which was more expressed at slower spinning speeds. This indicates a differential growth speed and a disordered base-layer. Once a degree of structural order is achieved, growth sets off at significantly higher rates than in the case of its surroundings.

In the 2MAT films (Fig.S2a,b), the RMS roughness of 7.86 and 6.55 nm was observed for the slow and fast recipe, respectively. This trend is similar to the one observed in PH-SAM. A more fibrous structure is observed for faster spinning speeds. In both cases, at higher altitudes fewer, less expressed craters are observed, yet they do not entirely disappear.

In the 10MAT films (Fig.S2c,d), the RMS roughness of 2.16 and 1.27 nm was observed for the slow and fast recipes, respectively. The same trend continues, which indicates a kinetically controlled formation of the initial layer. At later times, fast growth of the correctly conformed material sets in, which leads to small, segregated structures.

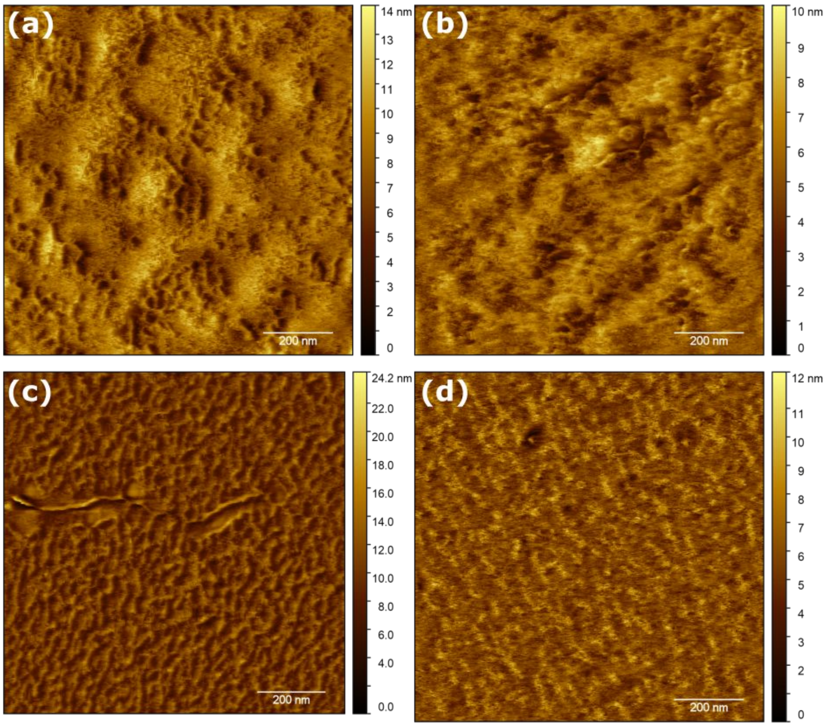

Figure S6: Bare Mica. 2MAT deposited via (a) slow and (b) fast recipe. 10MAT deposited at slow (c) and fast (d) recipe.

Bare glass

The amorphous nature of glass results in less organized, but more uniform material deposition (Fig.S3). Films are very smooth, similar to bare gold, yet the material is more disorganized, which results in frequent tip changes and a soft material (Fig.S4).

In the 2MAT films (Fig.S3a,b), the RMS roughness of 0.247 and 1.8 nm was observed for the slow and fast recipe, respectively. This indicates a deposition of material without a strong surface preference. In the case of the latter recipe, it was impossible to measure a full image without a tip-change, which reflects the poor attractive forces between material constituents.

In the 10MAT films (Fig.S3c,d), the RMS roughness of 0.467 and 0.503 nm was observed for the slow and fast recipe, respectively. At slower spinning speeds (Fig.S3c), distinct, elevated structures formed in a sea of disordered material. On the contrary to AD-SAM, we do not consider these to be poorly dissolved particles of PMAT-conglomerates from the mother-solution. This is because they are more frequent and more expressed in slower-spun films. Additionally, the few elevated features seen in the faster-spun films are different in character, i.e. they are less concise, larger, up to hundreds of nanometres in width, yet smaller in height.

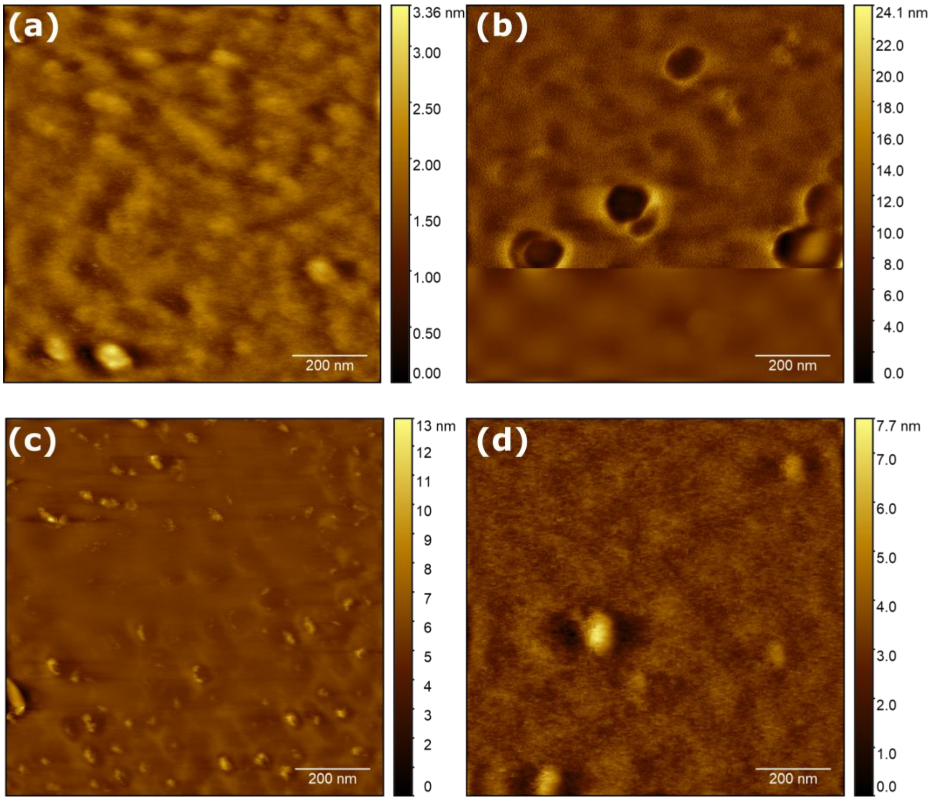

Figure S7: Bare glass. 2MAT deposited via (a) slow and (b) fast recipe. 10MAT deposited at slow (c) and fast (d) recipes.

Due to the more stable nature of slowly spun material, we focussed on the mechanical properties. This would be less prone to material desorption and tip-changes affecting the tip. The results can be seen in Fig. S4.

In the topography image (Fig.S4a), three elevated features in the lower part of the image surpass all other features in height – these can be easily identified when compared to the adhesion image (Fig.S4b). Therein, they appear as dark spots, *i.e.* they interact poorly with the tip and are self-contained. At a closer inspection, all elevated structures have poor adhesion to the tip, while depressions show the opposite trend.

Contrary to adhesion, the largest material deformation (Fig.S4c) was found for elevated structures – *i.e.* the larger the structure, the more flexible it becomes, as expected from a weakly-bound organic structure. The only difference from this trend is the dark feature in the lower left corner, which we interpret as an adventitious foreign particle, such as a glass particle introduced during substrate cutting. In dissipation, we are able to distinguish between elastic deformation (dark spots) and plastic, permanent deformation (bright spots). Therein, the true difference between larger and smaller structures comes to light, as the larger structures are mechanically more robust and only a limited amount of mechanical energy from the AFM-tip is dissipated in the particles.

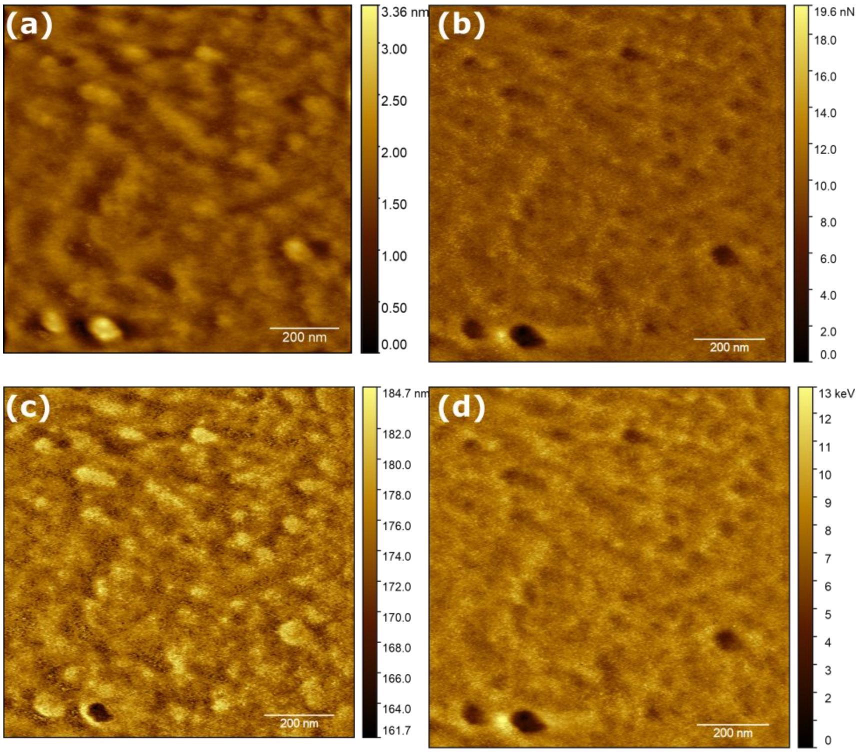

Figure S8: 2MAT on bare glass. (a) Topography, (b) adhesion, (c) deformation, (d) dissipation image of the same area. Colour scale of mechanical experiments in arbitrary units.
